# Role of NS2 specific RNA binding and phosphorylation in liquid−liquid phase separation and virus assembly

**DOI:** 10.1093/nar/gkac904

**Published:** 2022-10-19

**Authors:** Shah Kamranur Rahman, Khamal Kwesi Ampah, Polly Roy

**Affiliations:** Department of Infection Biology, London School of Hygiene and Tropical Medicine, London WC1E 7HT UK

## Abstract

Liquid−liquid phase separation (LLPS) has assumed a prominent role in biological cell systems, where it underpins the formation of subcellular compartments necessary for cell function. We investigated the underlying mechanism of LLPS in virus infected cells, where virus inclusion bodies are formed by an RNA-binding phosphoprotein (NS2) of Bluetongue virus to serve as sites for subviral particle assembly and virus maturation. We show that NS2 undergoes LLPS that is dependent on protein phosphorylation and RNA-binding and that LLPS occurrence is accompanied by a change in protein secondary structure. Site-directed mutagenesis identified two critical arginine residues in NS2 responsible for specific RNA binding and thus for NS2−RNA complex driven LLPS. Reverse genetics identified the same residues as essential for VIB assembly in infected cells and virus viability. Our findings suggest that a specific arginine−RNA interaction in the context of a phosphorylated state drives LLPS in this, and possibly other, virus infections.

## Introduction

In the past, many efforts have been put into understanding the mechanism of liquid-liquid phase separation (LLPS) in artificial biomimetic systems, but recently, there have been many studies on how LLPS may influence cellular activities. An increased interest in LLPS in natural system has revealed that LLPS are fundamental to most cellular processes and complex sets of phase transitions may underpin the formation of several subcellular compartments. LLPS may form small macromolecular condensates or more often form large subcellular compartments known as membrane less organelles (MLOs) (1–3).

Many viruses also develop their own, membrane-less compartments in the infected cells, where genome replication and virus assembly occur, which are referred to as ‘virusfactories’, ‘viroplasm’ or viral inclusion bodies (VIBs) (reviewed in (4)) (5–11). Several studies have demonstrated that these viral inclusion bodies (VIBs) adopt various liquid states (a condensed state or a dilute state) segregated by a phase separation in the cell cytoplasm similar to cellular MLOs. Both DNA and RNA viruses have been shown to utilize LLPS during their replication in host cells. For example, Rabies Negri bodies, the nucleocapsids of Measles virus, RSV and SARS-CoV-2 as well as Rotavirus viroplasm have all been shown to utilize LLPS for their assembly (6,12–15). However, the detailed mechanism of LLPS induced assembly and disassembly of membrane-less compartments such as VIBs in virus infected cells, is not yet clear.

Many transient LLPS structures are triggered by interactions between disordered regions of a protein and RNA (16–18) suggesting that virus encoded proteins with predicted regions of disorder may be part of an LLPS mechanism. To investigate this, we have studied VIB formation for Bluetongue virus (BTV), an Orbivirus within the *Reoviridae* family, where significant details of VIB assembly and disassembly are known (19–21). A single phosphorylated non-structural protein, NS2 is the principal component of virus factories and has the inherent property of forming membrane-less inclusion bodies when expressed in isolation (20,22). However, recombinant nonphosphorylated NS2 does not assemble into large VIBs although it still oligomerises to form decamers (9,23). Several reports have demonstrated that NS2 is responsible for interaction with, and recruitment into the VIBs of each of the newly synthesized BTV subcore proteins (VP1, VP3, VP4 and VP6) as well as the recruitment of viral ssRNA transcripts (9). Interaction with different RNA segments is achieved through several distinct RNA structures, which serve to recognize viral RNA from cellular RNAs within the infected cells (24). However, the phosphorylation status of NS2 neither enhanced nor reduced RNA binding activity in comparison to that of a nonphosphorylated NS2 (9). Within the virus factories, the assembled subcore acquires VP7, to form a stable core particle (9,25,26).

NS2 is not a component of the mature virus but is indispensable for the assembly of the primary replicase complex, which initiates secondary replication in the infected cell (26). The accumulating data on NS2 and its role in the replication cycle would be consistent with a VIBs formation mechanism that relied on gradual phase transitions of RNA-protein complexes, although formal evidence for this is currently lacking.

In this study, we have employed the most appropriate experimental approaches to investigate the role of LLPS in generation of BTV VPs formation and virus replication. The complex process of LLPS occurs through multivalent interactions driven by major factors such as phosphorylation, RNA binding, and intrinsically disordered region (IDR). Therefore, here we first studied these three factors individually *in vitro* under physiologically relevant conditions prior to confirming the combined effects in virus infected cells. Using a series of biophysical measurements, biochemical experiments, and biological assays, we show that NS2 forms ‘dilute’ and ‘condensed’ phases via an LLPS barrier and that kinase dependent NS2 phosphorylation and its RNA binding affinity regulate these phase separations. We elucidate the mechanism of RNA-NS2 condensate formation and identify the key interacting amino acid residues involved using site-directed mutagenesis. The effects of NS2 substitution mutants on *in vitro* protein-RNA interactions and *in vivo* virus survival studies using reverse genetics, showed that RNA binding to specific positively charged arginine residues in NS2 triggers VIB formation, wherein assembly and replication of newly synthesized BTV virus particles occur.

## Materials And Methods

### Cells and viruses

BSR cells (BHK-21 subclone) were maintained in Dul-becco’s modified Eagle’s medium (DMEM) supplemented with 5% fetal bovine serum (FBS) and grown at 35°C in 5% CO_2_. For the BS8 (BSR cells stably expressing NS2) cells, the media was additionally supplemented with the selection marker puromycin. For BTV infection, BSR or BS8 cell lines were used as previously described (23). For BTV mutant virus generation via reverse genetics, mutations were introduced into BTV-1 Segment 8 (S8, NS2) in the pUC19 plasmid backbone and viruses were generated as previously described (27). Insect cells, *Spodoptera frugiperda* (*Sf9*), were maintained in Insect-XPRESS™Medium and grown at 28°C.

### Cloning, protein expression and purification

NS2 mutant plasmids were generated via the QuikChange mutagenesis method (Agilent Technologies) and were inserted into the baculovirus transfer vector pAcYMl-S·tag for recombinant protein expression in insect cells. Phosphorylated NS2 proteins were expressed following the infection of *Sf*9 insect cells with recombinant baculoviruses at an MOI of 5, with cells harvested at 70 hours post-infection. Plasmid pET15b was used for non-phosphorylated NS2 expression in bacterial cell systems as previously described (23). NS2 proteins expressed in bacterial, or insect cells were purified using Ni-nitrilotriacetic acid (Ni-NTA) affinity purification and gel filtration, using a Superdex®200 10/300 GL (GE/Cytiva) column as previously described (23).

### *In vitro* phase separation of NS2

Recombinant protein was purified and concentrated to a stock concentration of 16 mg/ml. Tubes were prepared with increasing concentrations of proteins, ranging from 25 to 100 μM. Each protein concentration was then incubated in 20 mM Tris−HCl, pH 7.4 containing increasing salt concentrations: 25, 50, 100, 150 and 200 mM. The samples were centrifuged for 10 min at 14 000 RPM to remove any solid phase separated species which may have formed. For a direct measurement of phase separation in each reaction, the absorbance readings of a 200 μl reaction volume (0.5 ml tubes as cuvette) were measured at OD_350nm_ using the DS-11 nanodrop instrument (DeNovix). The threshold used was determined by observing multiple samples under the microscope and monitoring the OD_350nm_. Solid phase separations appeared as irregular shaped liquid droplets (as in Supplementary Figure S3) and had a higher OD_350nm_ value range (OD_350nm_ > 2−3), while liquid−liquid phase separated samples had circular droplets (as in Figure 5) and a lower OD_350nm_ value range (OD_350nm_ < 0.25−0.5). We adopted a figure between these ranges as the cut-off to ensure clear separation between true LLPS and aggregation. Any conditions that generate solid phase separation or resulted in precipitation in the samples were excluded in the phase boundary plots. The NS2−RNA (Segment 10) complexes were produced via the heating and snap cooling method. Briefly, a calculated amount of RNA was heated to 70°C prior to mixing with the NS2 protein sample on ice, where the complex was allowed to settle for at least 15 minutes.

### Visualization of *in vitro* NS2−RNA condensates and intracellular VIBs via microscopy

For bright-field microscopy visualization of phase separation, 10μl aliquots of NS2−RNA complexes were added to each well of a KOVA™ Glasstic™ Slide (Fischer Scientific) and were incubated at 37°C in a humid chamber for either 40−50 or 120 min, prior to analysis using an inverted microscope (ZEISS, Axio Vert.A1 KMAT). For visualization of NS2−RNA complexes via confocal microscopy (Zeiss Axiovert LSM 880 confocal microscope) the samples were stained for NS2 using an in-house guinea pig anti-NS2 primary antibody and an anti-guinea pig Alexa 488 coupled secondary antibody (Thermo Fisher Scientific). To visualize colocalization of RNA with NS2 in phase separated droplets, RNA was stained with GelRed®(Fisher Scientific). For VIB visualization in fixed cells, BSR cells grown on coverslips were infected with BTV (mutant or wild-type) at an MOI of 0.75 or 2, before fixing with 4% paraformaldehyde solution (Sigma-Aldrich/Merck) at 4h or 8h post-infection. Infected cells were then permeabilized with 0.5% Triton X-100 (Sigma/Merck) and blocked with 1% bovine serum albumin (BSA) (Sigma/Merck). Intracellular NS2 staining was carried out as previously stated and Hoechst 33342 (Thermo Fisher Scientific) was used for nuclei staining. For the 1,6-hexane diols (1,6HD) treatment experiments, BSR cells infected with BTV at an MOI of 2 were treated with 3.5% 1,6-hexane diols or polyethylene glycol 200 at 8h post-infection. After 8 min of treatment, cells were fixed and stained for NS2 prior to confocal microcopy analysis. For the 1,6HD removal assay, fresh media was exchanged after 8 min of treatment and the cells were then fixed after 22 min of the media exchange, stained for NS2 and analyzed via confocal microcopy. For live cells imaging, briefly, BSR cells were seeded in μ-Slide I Luer (ibidi ®, cat #80176) at a confluency of 40−50% and infected with green fluorescent BTV DISC strain at an MOI of 1−2 (28). Live or captured images of the stained samples were visualized using a confocal microscope with a ×20 objective. Zen software was used for data acquisition and image processing. Independent experiments were performed three times.

### Circular dichroism

The far-UV circular dichroism (CD) spectra of purified wtNS2 and the NS2 mutants were recorded in CD buffer (20 mM Tris, 100 mM NaCl, pH 7.4) at room temperature (20°C). For the RNA binding experiment, NS2 protein solutions (5.4 μM) at 4°C were aliquoted into four separate tubes at a volume of 150 μl prior to the CD reading. RNA segment 10 (S10) at 12 mg/ml was heated to 70°C before 0.1−2.0 μl of RNA solution was added to each NS2 tube (on ice), corresponding to a final RNA concentration of 0, 0.02, 0.26 and 0.46 μM RNA, respectively. The CD spectra of each tube containing RNA-NS2 complex was recorded along with that of RNA only samples of corresponding concentration. The spectra were collected in the 260−195 nm range with a 0.5 mm rectangular cell path length at 20°C, using a Chirascan & Chirascan Plus spectrometer (Applied Photophysics Leatherhead, UK) attached to a Peltier unit (Quantum NorthWest, UK). Each CD and UV spectra (for concentration measurements) were smoothed (window factor of 4, Savitzky-Golay method) and analysed using Origin V6 and APL Prodata Viewer v4.2.15 software. The spectra of the RNA−NS2 complexes with the varying RNA concentrations were normalized using a biosimilar chirality method at a single wavelength (208 nm) in mdeg (29). The plots were then converted to deg × cm^2^/dmol for representation (30).

### *In vitro* kinase assay with [γ^−^^32^P]−ATP

The *in vitro* kinase assay was performed on substrate protein NS2 (6X-His tag) using the kinase enzyme, Casein kinase 2 (CK2α) (GST tag); both were expressed separately in *E. coli* cells and purified using nickel or glutathione sepharose beads respectively. The kinase reaction was performed in a 50 μl reaction-mixture volume using 1X Tris reaction buffer (20 mM Tris, 100 mM NaCl, 1 mM DTT, pH 7.4) as described previously (23). Briefly, reaction tubes with a constant concentration of NS2 substrate at 2 μM, were incubated with an increasing concentration of CK2α, from 0.1 to 10 μM, and incubated at 37°C for 30 min. The kinase reaction was initiated by adding 0.5mM ATP, and after incubation the buffer was exchange to 1X Tris reaction buffer. The extent of phase separation was monitored through OD_350nm_ readings.

### Electrophoretic mobility shift assay (EMSA)

The RNA−NS2 complexes were prepared first by incubating viral RNA stocks of 1 μg/μl or 9 μg/μl at 70°C for 2 min. 1 μl of heated RNA was then added to NS2 at increasing concentrations, from 25 to 100 μM, on ice in binding buffer (2 mm MgCl_2_, 60 mm KCl, 150 mm NaCl, 20 mm HEPES, 1 mm EDTA, 1 mm dithiothreitol, 10% glycerol, pH 7.5 plus 1 unit of RNasin (Promega)) and incubated for 15 min to allow the complex to stabilize. The RNA−NS2 complexes formed were run onto a 0.8% agarose gel in TBE (Tris−borate−EDTA) supplemented with ethidium bromide at a concentration of 0.2 μg/ml and analysed using a gel imaging system G: BOX (Syngene). For competition assays, a 0.8 μM concentration of each wtNS2 or mutant protein, was added to 1 μg of heated S10 RNA in the presence of a 100-fold molar excess of tRNA. The resulting NS2−RNA complexes formed were analysed on 0.8% agarose gel in TBE (24,31).

## Results

### Non-phosphorylated NS2 forms RNA condensates

**Figure 1:**
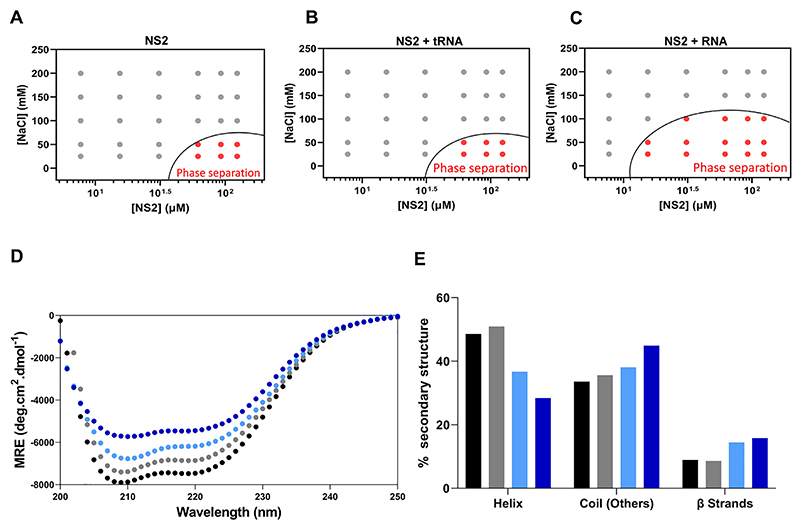
Phase separation plot of non-phosphorylated (P-)NS2 and Helix to coil and β sheet transition. NS2 at variable salt and protein concentrations. (**A**) Protein/NaCl concentrations plotted for optically significant phase separation (OD350_nm_). A phase boundary (black curve) drawn between positive phase separation score (red circles) and negative score (grey circles). (**B**) Phase boundary of (P-)NS2 in presence of denatured tRNA. (**C**) Phase boundary of (P-)NS2 in complex with viral RNA segment (S10). (**D**) Far-UV spectrum of NS2 apo alone (black circles ●), in the presence of 0.02 μM S10 (grey circles ●), in 0.26 μM S10 (cyan circles ●) and 0.46 μM S10 (blue circles ●). (**E**) The percentage secondary structure (helix, coil and β strands) estimations of Apo NS2 (black bar) and in presence of increasing concentrations of RNA segment S10. NS2 with 0.02 μM S10 (grey bar), with 0.26 μM S10 (cyan bar) and with 0.46 μM (blue bar).

To determine if NS2 alone could trigger phase separation *in vitro*, we first examined the influence of salt concentration on recombinant non-phosphorylated NS2 expressed in bacteria. When purified protein, treated with RNAse to remove any endogenous RNA, was incubated in increasing concentrations of NaCl (from 25 mM to 200 mM in Tris 20 mM, pH 7.4) and assessed by optical density (OD) at 350 nm, weak phase separation was evident only at low salt concentrations (~50 mM NaCl) (Figure 1A). The addition of a heat denatured nonspecific RNA (2 μM tRNAs) did not increase the level of condensate observed when compared with NS2 alone (Figure 1B). However, when preheated BTV segment 10 (S10) RNA, the smallest of the BTV RNA segments with 822 nucleotides, was added, it significantly enhanced condensate formation over a greater range of NaCl concentrations (Figure 1C). Thus, condensate formation by NS2 appears to depend on the binding of viral RNA.

The secondary structure of proteins, particularly IDRs play decisive mechanistic roles in phase separation (16,32,33). To assess if IDRs were responsible for the formation of the condensates observed with NS2−RNA complexes, we monitored changes in the secondary structure of 5.4 μM NS2 with increasing concentrations (from 0.02 to 0 0.46 μM) of S10 RNA by circular dichroism (CD). To minimize contribution of RNA we chose a concentration of RNA within a standard stoichiometry range (~56 kDa nucleoprotein spanning ~24 nucleotides) (24,34). Figure 1D shows the selected CD curves obtained at S10 RNA concentrations of 0, 0.02, 0.26 and 0.46 μM, respectively. The addition of S10 RNA caused a gradual increase in the proportion of coil (IDR) from 33.6% for ApoNS2 to 44.9% for NS2 bound with 0.46 μM S10 RNA segment (Figure 1E). Similarly, the beta sheet content increased from ~9% (ApoNS2) to 16% when complexed with viral RNA (Figure 1E). Thus, extensive secondary structure changes in both coil and β sheet correlate with the rise in condensate formation by BTV NS2 in the presence of viral RNA.

### Phosphorylation of NS2 regulates phase separation

Protein-RNA condensate formation has been shown to be regulated by protein phosphorylation (16,35). NS2 is the only BTV encoded phosphoprotein suggesting that phosphorylation could be associated with the observed RNA dependent phase separation.

**Figure 2:**
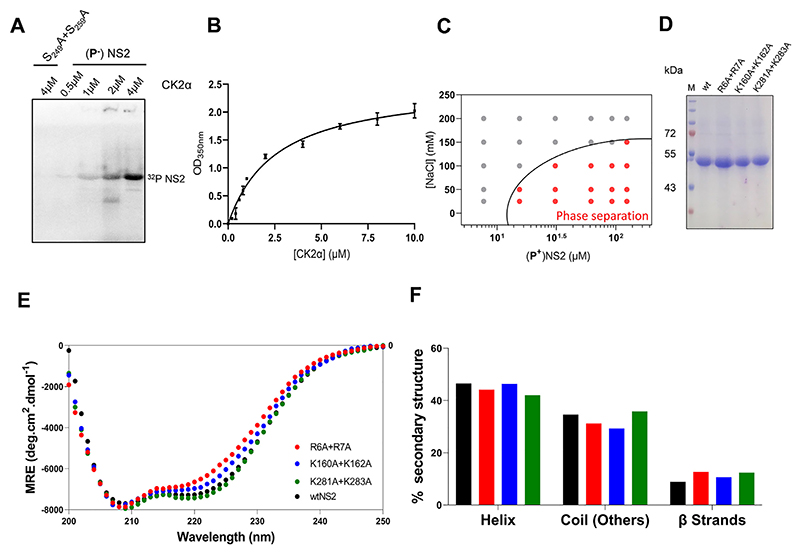
Phosphorylation regulates phase separation of NS2 and CD spectra comparison of phosphorylated wtNS2 and mutants (**A**) level of NS2 phosphorylation by CK2α kinase as determined by γ-32P signal intensity of NS2. For negative control, NS2 mutant deficit in phosphorylation serine residues (S249A + S259A) is substrate. Numbers above wells are molar concentrations of Kinase CK2α added in enzyme reactions with non-phosphorylated (P-)NS2 or mutant S249A + S259A as substrates. (**B**) Titration of CK2α concentration against phase separation of NS2 as a function of extent of phosphorylation, OD_350nm_. (**C**) Protein/NaCl concentrations plotted for optically significant phase separation of *in vitro* phosphorylated (**P^+^**) NS2. A phase boundary (black curve) drawn across between positive phase separation score (red circles) and negative score (grey circles). (**D**) SDS-PAGE gel after Coomassie blue staining showing wt and NS2 mutant proteins. M is the molecular mass markers. (**E**) Comparative far-UV CD spectra of wtNS2 (black circles) and mutants R_6_A + R_7_A (red circles), K_160_A + K_162_A (blue circles) and K_281_A + K_283_A (green circles). (**F**) Estimation of percent secondary structure contents of wt and mutant NS2 from far-UV CD spectra.

We incubated purified casein kinase II alpha (CK2α) at increasing concentrations (from 0.1 to 10 μM) with a constant concentration of NS2 (2 μM) to vary the phosphorylation state, validating each reaction by observing intensity of [^32^P]-NS2 produced. While there was no phosphorylation in negative control S249A + S259A mutant NS2 protein (9), the phosphorylation of NS2 increased with CK2α concentration in a dose dependent manner (Figure 2A), which further correlated with a gradual increase in the liquid phase separation of NS2 measured by OD_350nm_ (Figure 2B). Instead of relying on turbidity alone, we also observed liquid droplets under the confocal microscopy and ensured absence of solid aggregates (Supplementary Figure S1, supplementary material videos V1). Moreover, when *in vitro* phosphorylated NS2 was examined for salt-dependent phase separation, it showed a much higher tendency for the phase separation (Figure 2C) than the non-phosphorylated NS2 (Figure 1A). These data confirmed that phosphorylation of NS2 induces and regulates phase separation of NS2.

### Identification of RNA binding regions in NS2

NS2 recruits viral ssRNA preferentially but as there is no high-resolution structure of full-length NS2 or the NS2−RNA complex, we used computer-based programs to predict three putative RNA binding regions of NS2, (aa2−11), (aa153−166 and (aa274−286) (Supplementary Figure S2). These regions occur in the IDRs as determined by the PONDR output (Supplementary Figure S2) (36). Additionally, predicted structure models of NS2 using two different programs, I-Tasser and trRosetta (37,38) suggested that the NS2 molecule is extended with β sheet rich domains at both N- (aa1−169) and C-terminal (aa 266−354) regions, connected by a central disordered region (Supplementary Figure S2). The amino terminal region with two consecutive arginine residues (R6, R7) showed the highest localized disorder propensity. The predicted models are consistent with NS2 being able to readily change secondary structure through its central domain. The predictions also agreed with the crystal structure of the N-terminal domain of NS2, which showed disorder for R6, R7 and K160, K162 and missing electron density for aa 1-7 (PDB:1UTY) (39). Alanine substitution mutations for each charged residue were introduced as indicated in figures S2A and S2C and each mutant protein was expressed in insect cells in phosphorylated form. When analysed by gel electrophoresis (SDS-PAGE), all three mutant proteins, R_6_A + R_7_A, K_160_A + K_162_A and K_281_A + K_283_A were equivalent to the wild-type NS2 (wtNS2), in both size and stability (Figure 2D). We compared the CD spectra of each mutant with that of wtNS2 to assess the secondary structure changes (Figure 2E) associated with the mutagenesis and found no major changes in β strands (~10%), helix (~45%) or coil (~33%) (Figure 2F). Thus, alanine substitution of arginine or lysine did not result in substantial conformational change in the NS2 structure.

### Do the NS2 mutations at predicted RNA binding sites perturb BTV RNA binding specifically?

**Figure 3:**
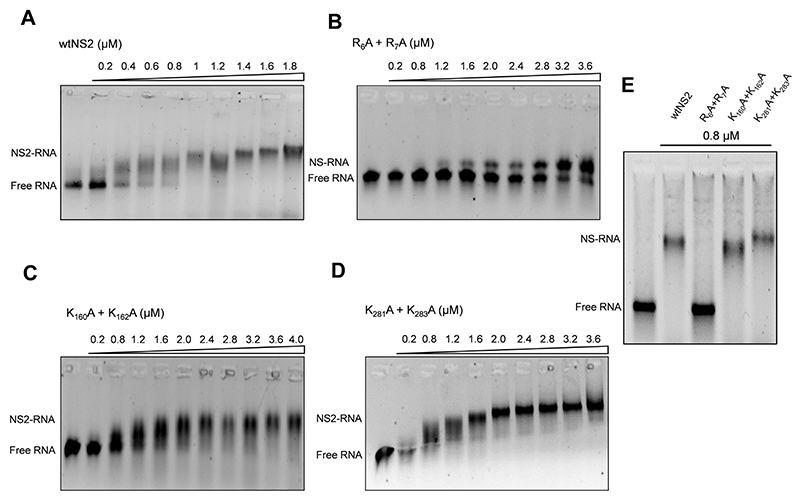
Mobility shift assay of RNA and (P^+^)NS2 protein complex. The numbers above lane from left to right denotes increasing concentrations of phosphorylated (**P^+^**)NS2 protein in micromolar range, μM. (**A**) The significant shift in viral S10 RNA in complex with wtNS2 on 0.8% agarose gel. Other panels show relative shifts in mutants R_6_A + R_7_A (**B**), K_160_A + K_162_A (**C**) and K_281_A + K_283_A (**D**), of which mutant R_6_A + R_7_A showed significant inhibition in mobility shift. (**E**) The competition binding of viral RNA S10 with wtNS2 or mutants RgA + R_7_A, K_160_A + K_162_A, K_281_A + K_283_A (0.8 μM) in molar excess of heat denatured tRNA.

To examine if the NS2 mutations described had any effect on binding to BTV ssRNA, S10 ssRNA (1.0 μg) was incubated with increasing amounts (from 0.2μM to 4μM)of each of the three mutant proteins alongside the recombinant wtNS2 as a control. Protein−RNA complexes were then analysed using an established electrophoretic mobility shift assay (EMSA) (24,31). Previously we showed that S10 adopts a complex stem loop structure that provides specificity for viral over cellular RNA when bound by NS2 (24,31). As expected, wtNS2−RNA complexes showed distinct retardation with an estimated dissociation constant, *K_D_*, of 0.91 (±0.08) μM demonstrating strong binding of RNA by wtNS2 (Figure 3A). In contrast, mutant R_6_A + R_7_A showed insubstantial complex formation, a *K*_D_ of ~2 (±0.1) μM while mutants K_160_A + K_162_A and K_281_A + K_283_A mutants retained RNA binding with a *K*_D_ of ~1.2 (±0.1) μM and 1.32 (±0.09) μM respectively (Figure 3B−D). Moreover, this specificity was maintained when the EMSA analysis was repeated in the presence of a 100-fold molar excess of non-specific yeast tRNA. The amount of S10 RNA bound to the wtNS2, K_160_A + K_162_A and K_281_A + K_283_A mutants was not affected by the presence of excess denatured tRNA, while mutant R_6_A + R_7_A showed a significant loss of S10 binding activity (Figure 3E). These data are consistent with S10 binding specifically to NS2 via the arginine residues at positions 6 and 7 in the N-terminal region of the protein while non-specific binding occurs to the lysine residues at 160/162 and 281/283.

### NS2−ssRNA complexes regulate phase separation

**Figure 4:**
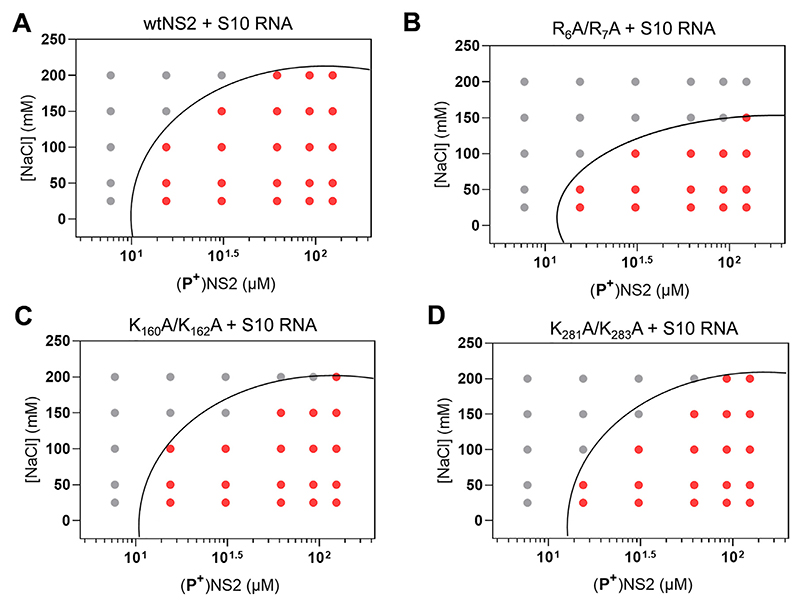
Phase separation plot of phosphorylated (P^+^) NS2 mutants in complex with RNA. Phase boundary (black curve) drawn between positive phase separation score (red circles) and negative score (grey circles) after incubation of the NS2−RNA complexes for 2 h. (**A**) Protein/NaCl concentrations of (**P^+^**) wtNS2 and S10 RNA complex. Phase plot of S10 RNA−protein complexes for mutants R_6_A + R_7_A (**B**), K_160_A + K_162_A (**C**) and K_281_A + K_283_A (**D**).

**Figure 5:**
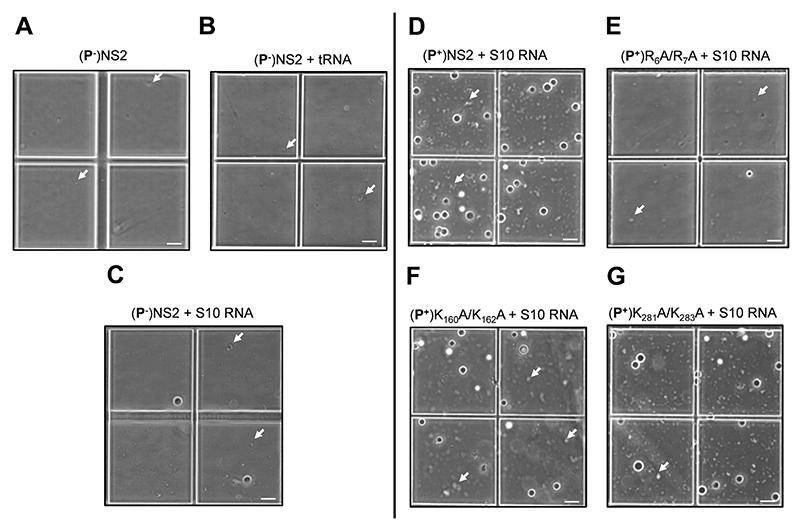
Visualisation of NS2 + S10 RNA condensates under phase contrast microscope. The left-hand side panels are phase separation condensates (visible as droplets) formed by mixing of RNA with non-phosphorylated (**P**^−^) NS2 and right-side panels with the phosphorylated (**P^+^**) wtNS2 and mutants. (**A**) Non-phosphorylated (**P**^−^)wtNS2. (**P**^−^)wtNS2 + denatured tRNA complex (**B**), (**P**^−^)NS2 + S10 RNA (**C**). Phase separation of S10 RNA + (**P^+^**)NS2 complexes for wtNS2 (**D**), mutant R_6_A + R_7_A (**E**), K_160_A + K_162_A (**F**) and K_281_A + K_283_A (**G**). The white arrow points to small condensates (minute droplets) apparent on close observation. Images are representative of three independent experiments. Scale bars, 40 μm.

To compare the effects of the mutant proteins on phase separation, each protein (25−100 μM) was incubated with S10 RNA for 1 h, and the resulting condensates were analysed at OD_350nm_. While nucleoprotein complexes for the mutants K_160_A + K_162_A and K_281_A + K_283_A showed a minor decrease in phase separation when compared to wtNS2, there was a significant loss of condensate formation for the R_6_A + R_7_A mutant, consistent with a role for this region in phase separation (Figure 4). Since LLPS is extremely sensitive to the charge distribution, we avoided any radical changes in NS2 such as, deletions of disordered regions or addition of large fluorescent/precipitation/solubility tags. The change observed in figure S3 was proportionate to a minor change in two amino acids, however, we show this small change is significant for phase separation. For a major change in logarithmic scale, we used a longer incubation period of 2 h (Figure 4). Phase contrast microscopy was used to directly visualize the NS2−RNA condensates. Large droplets, corresponding to the phase separated samples, were the majority observation under bright field view, in addition to some very small droplets (Figure 5A, white arrows). Non-phosphorylated NS2 sample alone (Figure 5A) or in the presence of with tRNA (Figure 5B) failed to demonstrate any phase separation while the non-phosphorylated NS2−S10 RNA complex showed a few large droplets indicating some phase separated NS2−RNA complexes (Figure 5C). However, a significant number of large droplets, with a tendency to coalesce, were apparent when BTV S10 was complexed with phosphorylated NS2 (Figure 5D). Further, when the mutant NS2 proteins and RNA were examined, only mutant R_6_A + R_7_A failed to demonstrate any significant LLPS (Figure 5E). Mutants, K_160_A + K_162_A and K_281_A + K_283_A, continued to demonstrate phase separation when bound to RNA S10 (Figure 5F, G).

**Figure 6:**
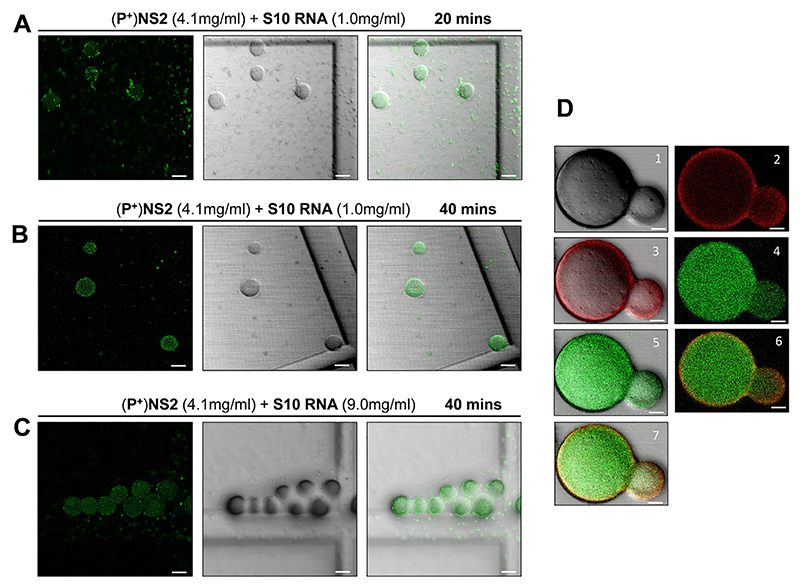
NS2 forms concentration dependent condensates. The left panel is green channel, middle is bright field and right panel is merged view. (**A**) purified phosphorylated NS2 and RNA S10 segment complex forms time dependent condensates. At 20 min, ~4.0 mg/ml (100 μM) NS2 (green) shows two populations at 20× magnification, small condensates (small green spots) and those coalescing into large condensates (circular drops). (**B**) After 40 min of incubation majority of NS2 population coalesced into large condensates. (**C**) At a higher concentration of RNA, these large condensates further fused to form associated assemblies. Images are representative of three independent experiments. Scale bars, 20 μm. (**D**) Viral RNA (S10, 1 mg/ml) stained with GelRed® is observed colocalising with NS2 that is stained with Alexa 488 (green fluorescence) in phase separated condensates, visible as large droplets after 40 min of incubation. Panel 1 is bright field (BF) view; 2 is red view; 3, red, BF merged view; 4, green view; 5 is green. Panel 6 is green, red merged view and 7 is green, red, BF merged view. Images are representative of three independent experiments. Scale bars, 5 μm.

**Figure 7:**
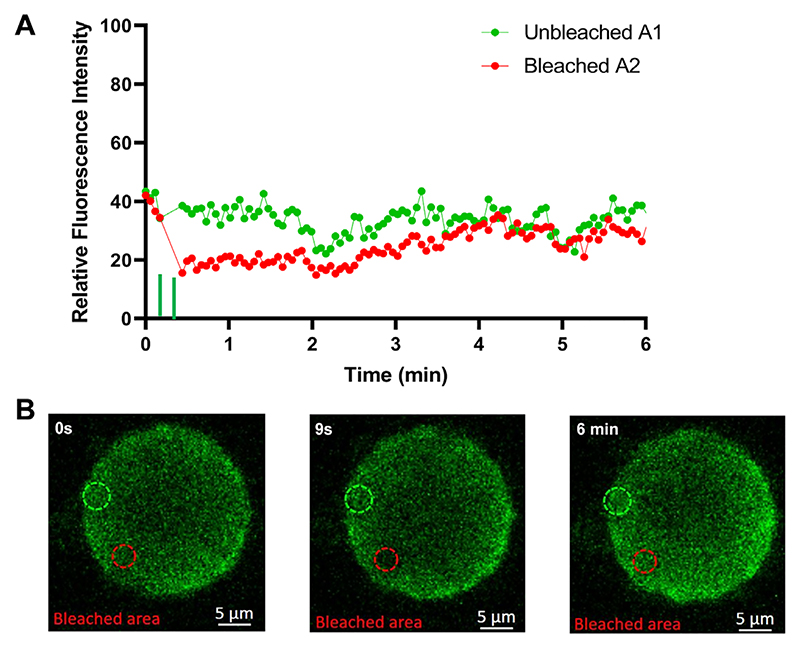
NS2−RNA complexes are in liquid phases. (**A**) The quantification of fluorescent intensity recovery after photobleaching from 0 s to 6 min. The green filled-circles represent datasets of intensity counts for the unbleached area A1, while the red filled-circles represent the bleached circle area (A2). The green vertical bars on the X-axis near 9 s denote bleaching doses. (**B**) The left, middle and right panels are snapshots of NS2−RNA complex droplets in liquid phases at 0 s (no bleaching), 9 s (bleaching) and 6 min (recovery after bleaching). The green fluorescence of NS2 was bleached in the area represented by the red circle (area A2) at 9 s (middle panel) showing final recovery at 6 min (right most panel). Scale bars, 5 μm.

**Figure 8:**
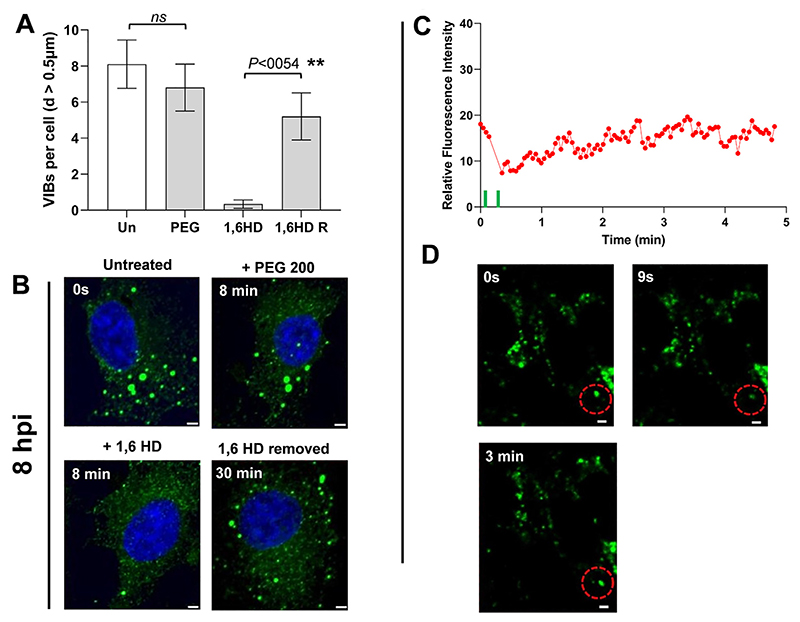
NS2−RNA condensates show liquidity in infected cells. (**A**) The average quantification of VIBs in infected cells with a diameter size of at least 0.5 μm. Un, untreated cells; PEG, PEG 200 treated cells; 1,6HD, 1,6-hexanediol treated cells and 1,6HD R, cells in fresh media with 1,6HD removed. (**B**) Representative confocal images of fixed cells, corresponding to the quantifications in A, at intervals of 8 min during 1,6HD and PEG treatment and 30 min after removal of 1,6HD. (**C**) The quantification of fluorescent intensity recovery in infected live cells after photobleaching of VIBs in bleaching area (red dotted circle), from 0 s to 5 min. (**D**) The top left and right panels show confocal snapshot images of unbleached VIBs (0 second) and just after bleaching (9 s) respectively. The lower panel image shows recovery at around 3 min after photobleaching. Scale bars, 2 μm.

To confirm that the observed droplets contained NS2−RNA complexes, we stained them with a fluorescent anti- NS2 antibody and visualized via confocal microscopy. When purified phosphorylated protein (~4 mg/ml) was incubated with S10 RNA (1.0 mg/ml) for 20 min, two populations of NS2 (green) were visualized, one forming large droplets, indicating phase separation, while the other population appeared as a scattering of very small granules (Figure 6A) whose number decreased with longer incubation times in favour of the larger forms, which became well-defined due to the even distribution of the NS2 (Figure 6B). We observed many smaller NS2 particles (green moving particles) and few very large droplets in liquid phases (supplementary material video V1). At higher RNA concentration (9 mg/ml), NS2 was essentially all found as large liquid droplets after 40 min of incubation (Figure 6C). The RNA component in the phase separated droplets was visualised with GelRed®and was visible only in large phase separated droplets appearing as a part of phase separated droplets with NS2 (Figure 6D, supplementary material video V2). These data are consistent with that obtained earlier (Figure 1 and 3) and confirmed that phase separated droplets contained NS2−RNA complexes. The liquid nature of droplets was also tested with Fluorescence recovery after photobleaching, FRAP (Figure 7). While the control area (A1, indicated by dotted green circle) in the liquid droplets remained unbleached, the bleached area (red circle A2) at 9s, started recovery soon after photobleaching, with stabilising after 2−3 min, and confirming liquid nature of NS2−RNA complex in these droplets (Figure 7A, B, supplementary material video V1). The liquid nature of NS2−RNA condensates was also validated in cells infected with the virus. Infected cells in presence of 1,6-hexane diol (1,6HD), a compound known to dissolve liquid−liquid phase separated condensates, showed significant reduction in VIB formation, in contrast to the infected cells treated with polyethylene glycol which does not affect condensates (control, Figure 8A). Further, when media of the 1,6HD-treated cells was replaced with fresh media, its dissolution effect was lost rapidly and VIBs formation was recovered gradually as observed by confocal microscopy (Figure 8B). The liquid nature of VIBs was further examined in infected cells with green fluorescent BTV DISC (disabled infectious single cycle) strain (28) for live cell imaging followed by FRAP analysis (Figure 8C, D). The recovery of the VIBs (green fluorescence) in bleached area could easily be identified, consistent with the data obtained with the 1,6 HD-treated cells (Figure 8C, supplementary material video V3).

### Disruption of RNA binding residues in NS2 inhibited virus replication

**Figure 9:**
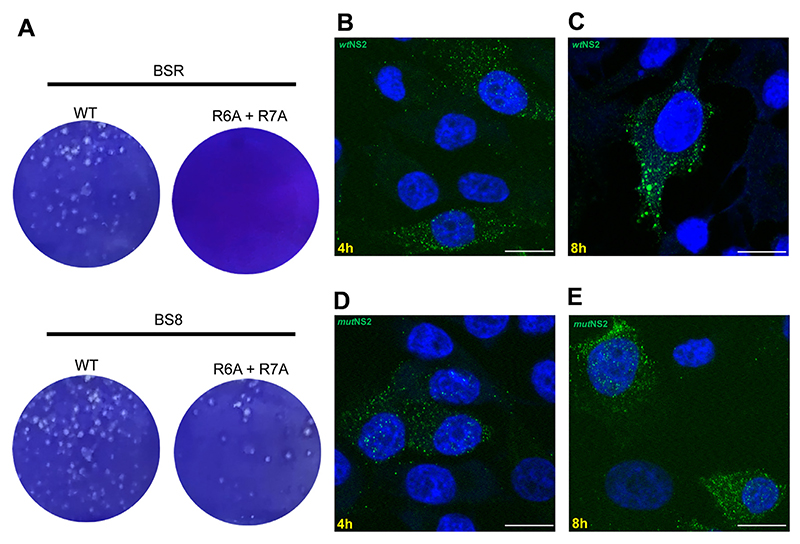
Disruption of arginine residues in NS2 affects plaque formation and VIB visualization. (**A**) BSR and BS8 cell monolayers were transfected with wt and R6A + R7A mutant S8 together with the 9 RNA segments. Images show plaques formed by recovered viruses in BSR and BS8 (BSR cells expressing NS2) complementary cells. (**B**) NS2 expression at 4 h post-infection of BSR cells with wt virus. (**C**) Matured VIBs formed of NS2 after 8 h post-infection. (**D**) NS2 expressions in cells infected with mutant virus (R_6_A + R_7_A) at 4 h and 8 h post-infection (**E**) showing absence of matured VIBs. Images are representative of three independent experiments. Scale bars, 10 μm.

To determine whether RNA−NS2 complex formation and phase separation are essential for virus replication, the R6A + R7A mutations in the NS2 gene were assessed for virus recovery using reverse genetics (40). When BTV susceptible BSR cells were transfected with a mutant S8 (encoding NS2 R6A + R7A protein) together with the 9 wt genome segments, no virus could be recovered, in contrast to cells transfected with all 10 wt segments where virus recovery was observed. To confirm that the loss of virus recovery with the mutant genome was due to the R_6_ and R_7_ mutations, virus recovery was performed using a novel complementary cell line of BS8 cells expressing an incorporated wtNS2 protein. Both mutant and wt viruses were recovered at the comparable level and phenotype (Figure 9A). However, passage of the mutant virus was only possible on the complementary cell line while in BSR cells only the wt virus grew. In addition, when the formation of VIBs was visualized at the optimized early time points of 4hr and 8hr post infection (h.p.i) by confocal microscopy, NS2 was visible in native BSR cells which had, by 8hr, started to form large VIBs (Figure 9B, C) (41). In contrast, in mutant virus infected BSR cells, even at 8 hpi, larger VIBs were absent and there was little visible difference between the 4hr and 8hr time points (Figure 9D, E). These data confirm that the lack of NS2−RNA complex parallels phase separation *in vivo* and is essential for virus replication.

## Discussion

Over the last two decades many studies have focused on virus inclusion bodies which are essential for the accumulation of virus components, genome packaging and assembly of progeny virions (4−8,42). Infection of cells by BTV produces prominent VIBs which appear initially as granular material scattered throughout the cytoplasm, but then coalesce to form prominent globular perinuclear inclusion bodies (aka condensates) (9−11). VIBs lack membranes and their dynamic presentation is akin to liquid−liquid phase transitions which typically condense RNA and protein into defined structures (reviewed in (43)). VIB formation is driven by a single non-structural protein, NS2, which is rich in positively charged residues and has affinity for viral ssRNA over host RNAs (24,31). It is also phosphorylated by the host CK2α, and phosphorylation is related to the level of aggregation although is not necessary for RNA-binding (9). Using inhibitors, specific mutations in the viral genome and confocal microscopy, previous data showed that CK2α activity is important for BTV replication (11). With cellular protein phosphatase 2A, CK2α regulates the dynamic nature of VIB assembly/disassembly, and in consequence, virus assembly and replication.

An outstanding question has been how a single protein is able to capture all the virion components and produce an environment conducive to virus assembly. NS2 is disordered and a three-dimension structure for the entire molecule is lacking. A structure has been solved for an N-terminal fragment (aa 8−160) which is consistent with a role in RNA binding and oligomerisation but fails to explain any other ascribed NS2 function (39). An increased acknowledgement of the ability of disordered proteins, notably RNA binding proteins, to undergo liquid−liquid phase transitions (44) prompted us to re-examine NS2 for its ability to behave similarly as a plausible explanation of its role in VIB formation and function. A recent study of rotavirus has shown that VIBs or ‘virus factories’ are indeed LLPS structures and that these are triggered by interactions of two different viral non-structural proteins, NSP2, an RNA binding protein and NSP5, a phosphoprotein (6) but the direct role of each protein was not delineated, or if cellular processes, such as cytoskeleton remodeling, kinase or calcium signaling responses, were involved. In this investigation, we demonstrate that the phosphorylated state of NS2 leads to phase separation of condensates in a dose dependent manner consistent with our previous studies that non-phosphorylated NS2 fails to assemble into VIBs (9). In addition, the presence of BTV RNA, but not competitor, boosted LLPS formation consistent with our mapping a unique hairpin-loop secondary structure in BTV RNA (24,31,45,46), which provides specificity for how BTV mRNAs are selected from the pool of cellular mRNAs for incorporation into assembling virus particles. Our data demonstrate that RNA complexes with NS2 are an additional trigger for LLPS formation and suggests that this may be the mechanism by which cellular molecules are efficiently excluded from the site of assembly.

Using an electrophoretic mobility shift assay (EMSA) specific RNA binding was mapped to Arg 6 and 7 with no apparent role for lysines in the central and C-terminal domains (47). The guanidinium group in arginine residues can simultaneously form multivalent co-ordinations with many RNA groups (through H-bonds and pi stacking), which stabilize protein-RNA complexes (48) and induce phase separation (49). LLPS is exceptionally sensitive to even minor changes in the charge or conformation of macromolecules (50) and it seems probable that RNA binding to the stable N-terminal structure of NS2 initiates conformational change in the disordered central domain may facilitate further intermolecular interactions resulting in phase separation (reviewed in (44)). Consistent with this, CD data showed helix-to-coil transitions in NS2 concomitant with RNA binding. For a largely unstructured protein such as NS2 there may be some deviation between the theoretical calculation of secondary structure from a predicted structure and that obtained from an experimental CD curve. Particularly it is important to note that the theoretical secondary structure is predicted for monomeric NS2, while the experimental CD calculation is for NS2 oligomers (mainly decamers), which may contribute to a crowding effect on protein folding.

To confirm these interactions as essential for virus replication, we exploited BTV reverse genetics to generate a mutant virus in which the key arginine resides were changed to alanine. Alanine substitutions are minimally disruptive for the other NS2 functions, decamer formation (23), NTPase activity (51) and Ca^2+^ sensing (23). Rescue of the wild type or mutant virus was only possible in a NS2 complementing cell line and when passaged onto normal BSR cells the mutant virus failed to assemble VIBs of any significance, in keeping with the loss of LLPS function. The demonstration of an *in vivo* loss of function with mutations deduced from *in vitro* studies to moderate a biophysical process provides a strong endorsement for the role of a specific arginine−RNA interaction and LLPS formation in the virus replication cycle. The phenotypes at early time points are consistent with the rationale that failure of VIBs formation was due to inhibition in RNA binding activity of NS2. The longer the duration of virus replication, the more are chances of dampening of phenotype effect of mutants under investigation. Moreover, there could be other possible effects of cellular or viral factors. A longer duration of infection (e.g. 24 hpi) or multiple virus replication cycle was therefore avoided. Broadly, our findings are consistent with other reports, suggesting that LLPS in different viruses, even in unrelated virus families, primarily depends on three factors: an IDR, phosphorylation and RNA binding (16,52). BTV NS2 has all these functions, in addition to which we emphasize here the importance of arginine−RNA interaction for phase separation in the cellular environment. Recently, protein-RNA interaction has been suggested as a valid drug target in SARS-CoV-2 RNA binding protein (53) and in light of our data, we suggest that targeting LLPS formation is a valid pharmacological target to inhibit VIBs assembly and consequently, virus replication.

## Supplementary Material

Supplementary Material

## Data Availability

All data generated or analyzed during this study are included in this published article (and its supplementary information files). The NS2 predicted model is available from the corresponding author or the direct download link (BTV_NS2.pdb).
