# Supplementary material for: Role of NS2 specific RNA binding and phosphorylation in liquid−liquid phase separation and virus assembly
Source: Nucleic Acids Res. Author manuscript; Available in PMC 2022 Nov 7. (PMC9638936; doi:10.1093/nar/gkac904)
Supplement: Supplementary Material [file EMS155994-supplement-Supplementary_Material.zip › Legends to videos NAR-02630-R-2022.R1.docx]

**Manuscript ID:** *NAR-02630-R-2022.R1*

**Legends to videos**

**Supplemntary video v1:** FRAP analysis of the NS2-RNA complex *in vitro*. The NS2 molecules stained with green fluorescence within the spherical phase separated droplet are dynamic. The measurement of changes in the fluorescence intensity is plotted in Figure 7.

**Supplemntary video v2:** Z-stack visulaization of NS2-RNA complex as phase separated droplets. The viral RNA (S10, 1mg/mL) stained with GelRed® colocalises with NS2 protein molecules that are stained with Alexa 488 (green fluorescence). The representative snapshot images are shown in Figure 6D.

**Supplemntary video v3:** FRAP analysis of VIBs in virus-infected cells confirms the liquid nature of VIBs (green fluorescence). The measurement of changes in the fluorescence intensity is plotted in Figure 8C.
