# Supplementary material for: Role of NS2 specific RNA binding and phosphorylation in liquid−liquid phase separation and virus assembly
Source: Nucleic Acids Res. Author manuscript; Available in PMC 2022 Nov 7. (PMC9638936; doi:10.1093/nar/gkac904)
Supplement: Supplementary Material [file EMS155994-supplement-Supplementary_Material.zip › Supplementary material Sept 26.docx]

**Extended figures**

**
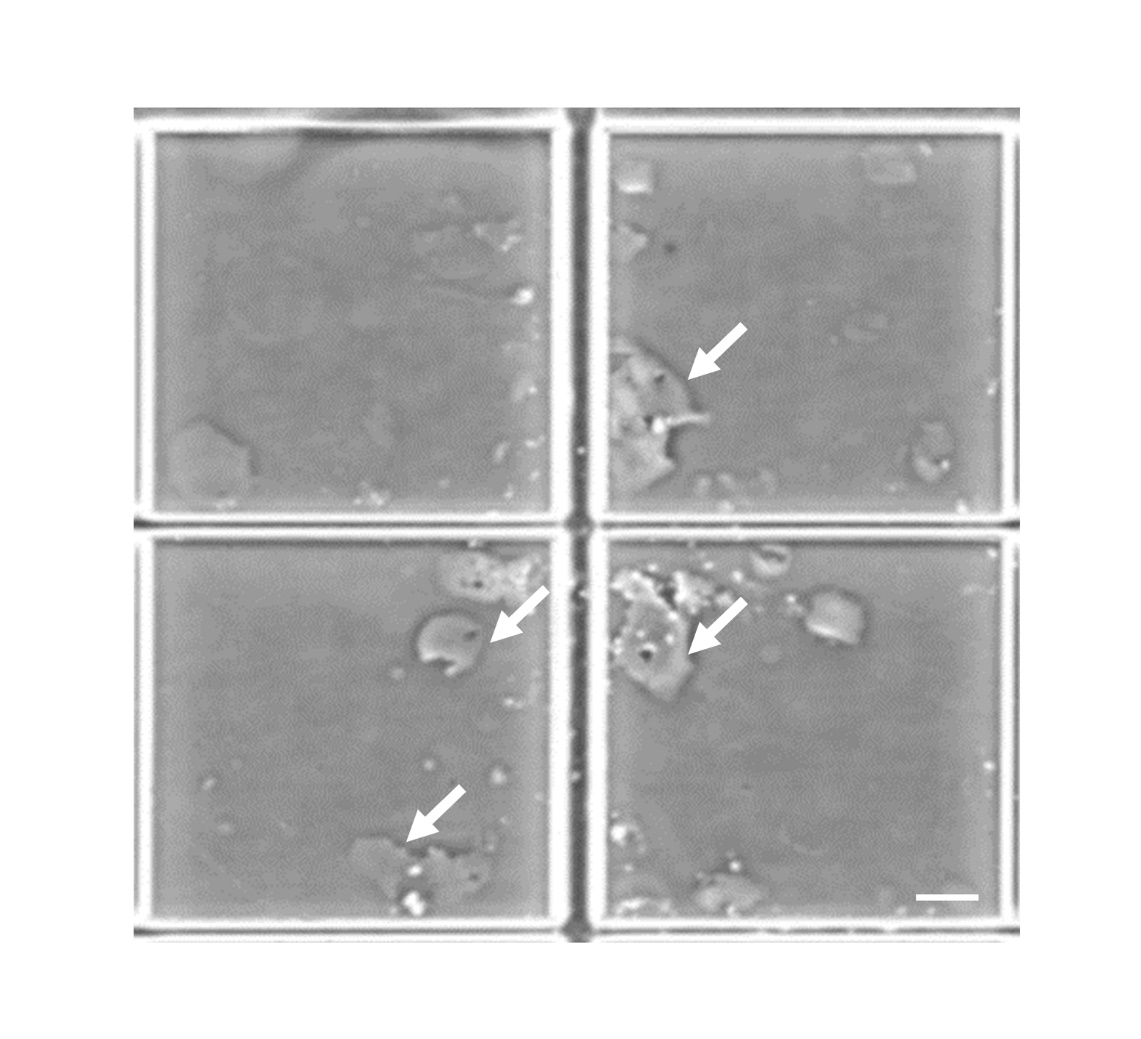
**

**Supplementary Figure 1: Visualization of solid aggregates under bright field microscopy.** The figure shows presence of irregular shaped solid aggregates exclusively (white arrow) with OD_350nm_ >2.0. Scale, 40 µm.

**
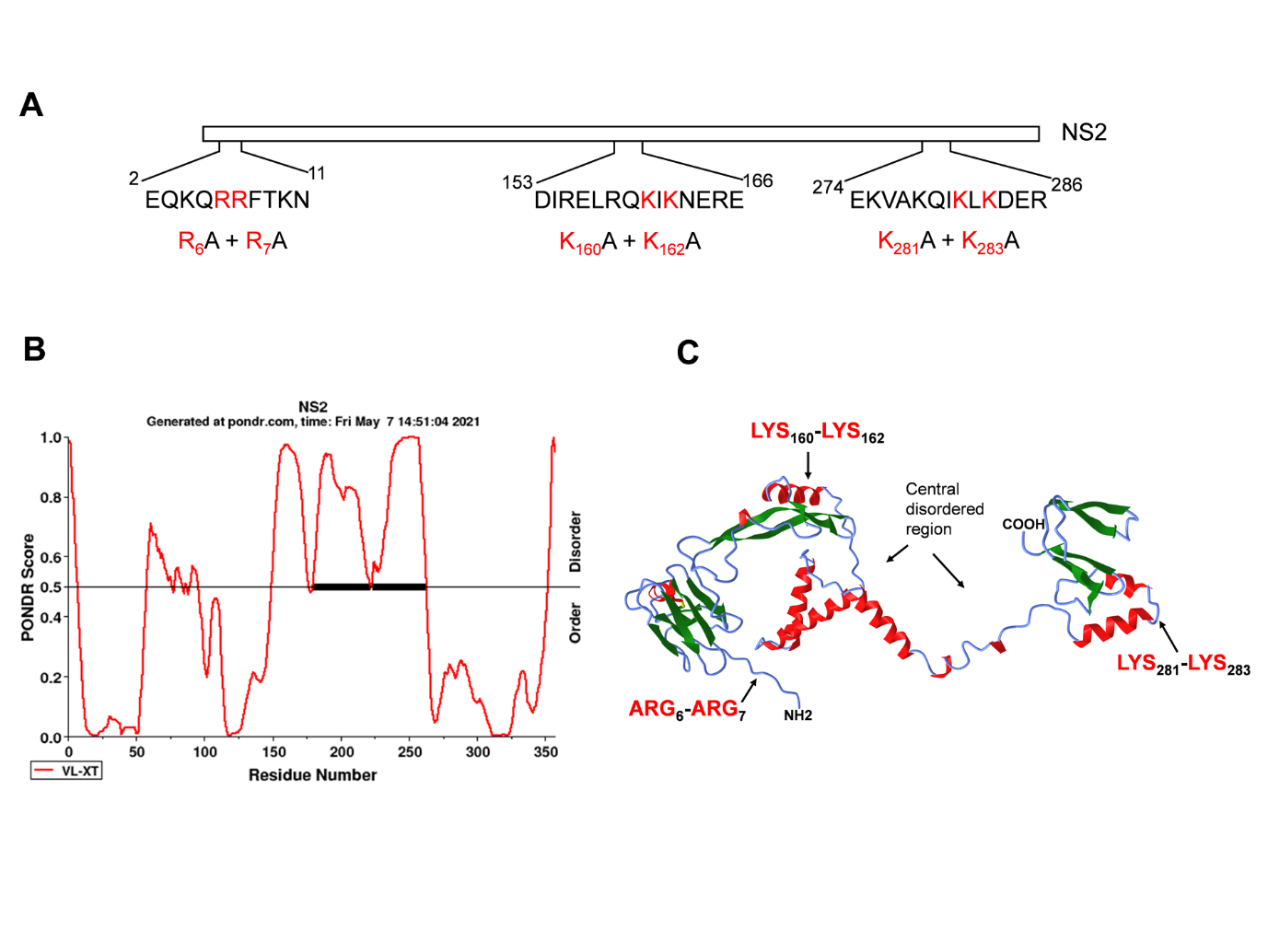
**

**Supplementary Figure 2: Identification of IDR and putative RNA binding regions in NS2 (A)** NS2 full-length representation showing three regions, amino acids aa 2-11, aa 153-166 and aa 274-286 with higher theoretical disorder propensities. The putative RNA binding residues (arginine, **R** and lysine, **K**) and the positions of alanine mutations introduced are indicated. **(B)** PONDR plot showing disordered regions in full-length NS2, the probability scores higher than 0.5 is with disorder propensity. **(C)** The model of full-length NS2 predicted by trRosetta showing an extended molecule with β sheet rich domains in the both N-terminus, residues 1-169 and C-terminus, residues 266-354 connected by a central disordered region consisting of 3 putative α helices and coil. The lysine and arginine residues are indicated. The model suggests a molecule that can undergo significant secondary structural changes.

**
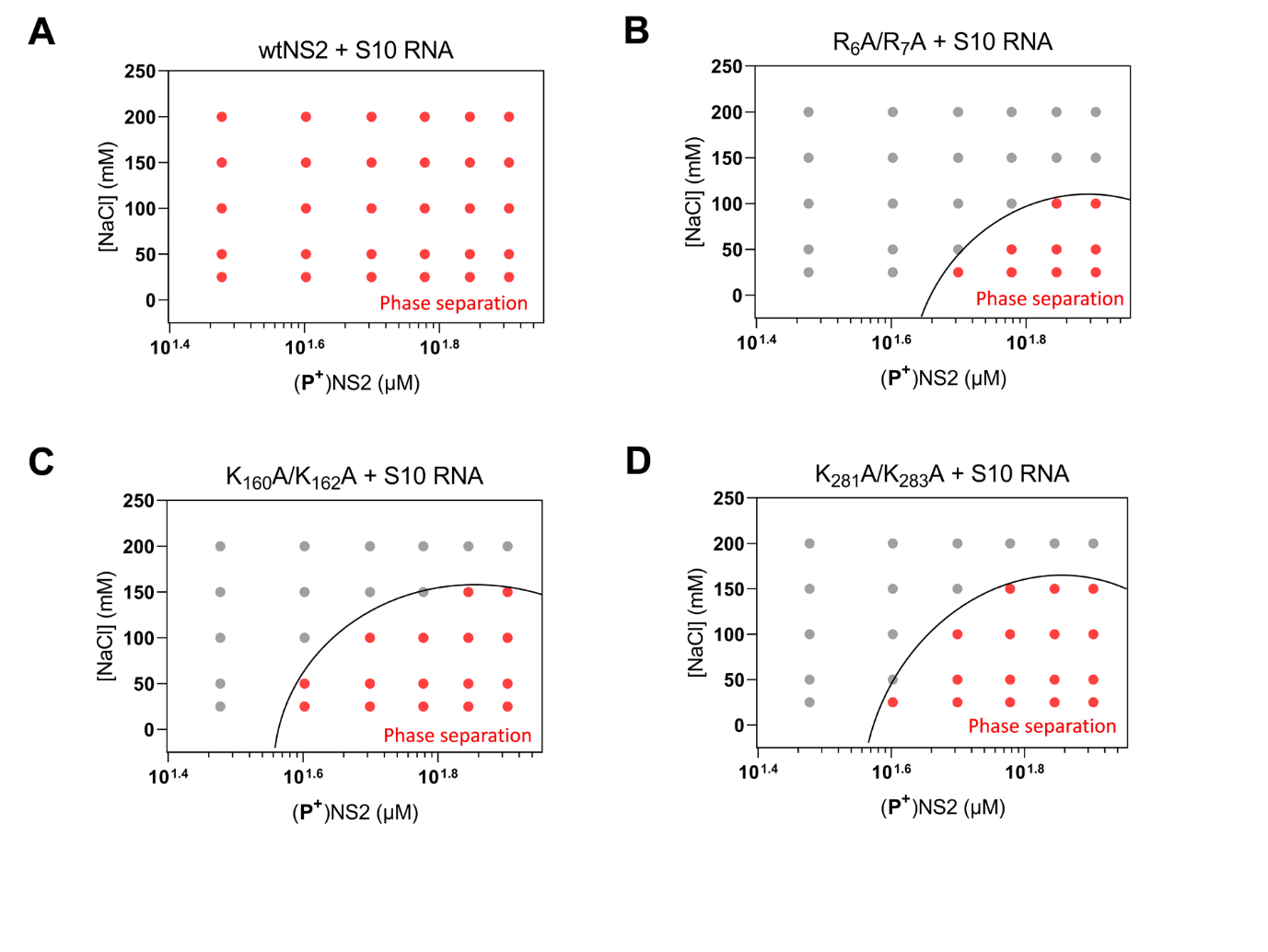
**

**Supplementary Figure 3: Phase separation plot of phosphorylated** (**P^+^**) **NS2 mutants with RNA**. Phase boundary (black curve) delineating positive phase separation score (red circles) and negative score (grey circles) after incubation of NS2-RNA complex for 40 minutes to 1-hour **(A)** Protein/NaCl concentrations of (**P^+^**) wtNS2 and S10 RNA complex. Phase plot of S10 RNA protein complexes for mutants R_6_A+R_7_A **(B),** K_160_A+K_162_A **(C)** and K_281_A + K_283_A **(D)**.
